# Changes in rural caregivers' health behaviors while supporting someone with cancer: A qualitative study

**DOI:** 10.1002/cam4.7157

**Published:** 2024-04-04

**Authors:** Elizabeth A. Johnston, Katelyn E. Collins, Jazmin N. Vicario, Chris Sibthorpe, Michael J. Ireland, Belinda C. Goodwin

**Affiliations:** ^1^ Cancer Council Queensland Fortitude Valley Queensland Australia; ^2^ School of Exercise and Nutrition Sciences Queensland University of Technology Kelvin Grove Queensland Australia; ^3^ Population Health Program QIMR Berghofer Medical Research Institute Herston Queensland Australia; ^4^ School of Psychology and Wellbeing University of Southern Queensland Springfield Queensland Australia; ^5^ Centre for Health Research University of Southern Queensland Springfield Queensland Australia; ^6^ School of Population and Global Health University of Melbourne Melbourne Victoria Australia

**Keywords:** alcohol, behavior change, diet, oncology, physical activity, supportive care

## Abstract

**Purpose:**

Caring for someone with cancer has a significant impact on usual routines, including caregivers' ability to maintain their own health and wellbeing. Caregivers living in rural areas face additional challenges in supporting someone with cancer, and little is known about the impact of caregiving on the health behaviors of rural caregivers. Therefore, this study explored how caring for someone with cancer affected rural caregivers' health behaviors.

**Methods:**

Through semi‐structured interviews, 20 rural caregivers described changes in their health behaviors while caring for someone with cancer and the factors underlying these changes. Specific prompts were provided for diet, physical activity, alcohol, smoking, sleep, social connection and leisure, and accessing health care when needed. Interviews were audio‐recorded and transcribed verbatim. Content analysis was used to identify changes in health behaviors and the factors underlying these changes. The factors identified were mapped to the socioecological framework, identifying areas for intervention across multiple levels (individual, interpersonal, organizational, community, and policy).

**Results:**

Rural caregivers reported both positive and negative changes to their diet, physical activity, alcohol, and smoking. Sleep, social connection and leisure, and accessing health care were negatively impacted since becoming a caregiver.

**Conclusions:**

Designing interventions to address rural caregivers' coping strategies, reduce carer burden and fatigue, improve access to cooking and exercise facilities and social support while away from home, reduce the need to travel for treatment, and increase the financial support available could yield widespread benefits for supporting the health and wellbeing of rural caregivers.

## Graphical abstract

This study examined changes in rural caregivers' health behaviors while caring for someone with cancer and the factors underlying these changes, through qualitative interviews. Rural caregivers reported both positive and negative changes to their diet, physical activity, alcohol, and smoking, while sleep, social connection and leisure, and accessing health care were negatively impacted since becoming a caregiver. Designing interventions to address rural caregivers' coping strategies, reduce carer burden and fatigue, improve access to cooking and exercise facilities and social support while away from home, reduce the need to travel for treatment, and increase the financial support available could yield widespread benefits for supporting the health and wellbeing of rural caregivers.

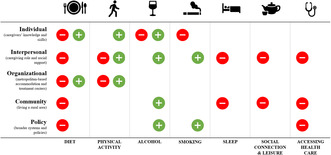

## 1 INTRODUCTION

Cancer is a common health condition worldwide, with an estimated 20 million new cases diagnosed in 2022, and this number is expected to increase by 77% by 2050.^1^ In Australia alone, one in two people will be diagnosed by age 85.^2^ With over 90% of cancer survivors being supported by an informal caregiver (i.e., family member or close friend), a large proportion of the Australian population will care for a loved one with cancer during their lifetime.^3^ The support provided to cancer survivors by informal caregivers (hereafter, ‘caregivers’) is vital, with much of the cancer survivors' symptom management occurring at home, outside of healthcare settings.^4^ Caregivers may also provide transport to and from medical appointments, emotional and psychosocial support, and practical assistance with activities of daily living.^5^, ^6^, ^7^ This direct care for cancer survivors involves, on average, more than 40 h per week, with the caregiving role described as a “full‐time job.”^8^, ^9^ Further, the presence of a caregiver has been linked to improved outcomes for cancer survivors, including their physical, functional, and emotional wellbeing.^10^

Given caregivers receive little to no preparation for this role^11^, ^12^ and few interventions include support for cancer caregivers,^13^, ^14^ it is unsurprising that caregivers experience elevated anxiety and depression and significant unmet needs for information and support.^15^, ^16^, ^17^ In patient‐caregiver dyads, the emotional distress experienced by the caregiver has been shown to be as high as, or even higher than, the patient's level of distress.^18^, ^19^ While some studies report improvement in health behaviors since becoming a caregiver,^20^, ^21^ there is evidence that caregiving is also associated with poorer diet quality, reduced physical activity, increased cigarette use, and reduced sleep duration.^21^, ^22^, ^23^ Poorer health and wellbeing from fulfilling the caregiver role can increase caregiver burden, compromising caregivers' ability to provide their vital care.^24^

Caregivers living outside of major cities face additional challenges in their caregiving role. In Australia, an estimated 28% of the population resides in a ‘rural’ area,^25^ referring to anywhere outside of major cities, including inner and outer regional, remote, and very remote areas.^26^ Compared to their urban counterparts, rural caregivers must navigate longer travel distances and associated costs for the patient to access treatment.^27^, ^28^, ^29^ Many rural caregivers also experience disruptions to employment and financial stress while caring for someone with cancer.^30^, ^31^ Further, research shows that rural caregivers are more likely to experience poorer mental health‐related quality of life than age‐matched counterparts in the general population,^18^ and many report unmet needs for information to manage their own health and wellbeing.^32^ Despite this, little is known about how caring for someone with cancer affects the health behaviors of rural caregivers.

Therefore, this study explored how caring for someone with cancer affected rural caregivers' health behaviors. The research questions guiding this investigation were: (1) what changes in health behaviors do rural caregivers experience while caring for someone with cancer, and (2) what are the socioecological factors underlying these changes? Insights from this study can contribute to a better understanding of how caring for someone with cancer impacts caregivers' health behaviors and how to support the health and wellbeing of rural caregivers.

## 2 METHODS

### 2.1 Participants and recruitment

Adults (aged 18 years or older) who self‐identified as providing informal support to someone with a cancer diagnosis, could speak and understand English, and were able to provide informed consent were eligible for this study. Twenty rural caregivers were recruited for this study from various avenues. Eleven participants were guests at one of the Cancer Council Queensland (CCQ) accommodation lodges recruited between June 2022 and June 2023. CCQ lodges are located in five major cities in Queensland and offer subsidized accommodation to cancer patients and their caregivers traveling more than 50 kilometers for cancer treatment. A cancer support advisor informed eligible guests of the study and with their permission, passed their contact details on to the research team. Eighteen rural caregivers from another longitudinal study led by the research team^33^ who were caring for someone who had undergone cancer treatment or follow‐up in the last 12 months were invited to this study, with six completing an interview. One participant was recruited by approaching group administrators of private Facebook cancer support groups to ask if they would share a post about the study. The remaining two participants were recruited via word‐of‐mouth. Reasons for not participating in this study were feeling overwhelmed or the patient's latest treatment (or follow‐up) occurring more than 12 months ago. This study was approved by the Human Research Ethics Committee of the University of Southern Queensland (H17REA152). All participants provided informed consent (written and/or verbal). This study is reported using the Standards for Reporting Qualitative Research (see Table S1).^34^

### 2.2 Data collection

A qualitative approach was chosen to capture rich and detailed accounts of participants' experiences in their own words. Individual interviews were conducted with rural caregivers using a semi‐structured guide (see Table S2) developed by the research team. Open‐ended questions were drafted based on the study aim to investigate how caring for someone with cancer affects the health behaviors of cancer caregivers. The draft interview guide was pilot tested with two caregivers known to the research team, whose data was not included in this analysis. Following pilot testing, prompts for specific health behaviors were included in the final interview guide, as caregivers' found it easier to reflect on specific behaviors rather than their health and wellbeing in general. These prompts were for caregivers' diet, physical activity, smoking, alcohol, sleep, social connection and leisure, and accessing health care when needed, based on the most recent systematic review on this topic.^35^ To describe the study sample, the interview included prompts for relationship to the person with cancer, time since their most recent cancer diagnosis, cancer type, caregiver age, and postcode of residence. Postcode of residence was used to determine level of geographical remoteness using the Australian Statistical Geography Standard Remoteness Structure.

Before conducting the interviews via telephone, the first author underwent training for speaking with distressed research participants and referring them to appropriate support. The first author had no prior relationship with participants. The average interview duration was 30 min (range 12–52 min), with a total of 10.5 h recorded. Audio recordings were transcribed verbatim using Microsoft Teams.

### 2.3 Data analysis

Participant characteristics were extracted from transcripts and summarized using descriptive statistics. Content analysis was used to identify and describe changes in health behaviors since becoming a caregiver and factors underlying these changes. Content analysis is a qualitative approach that involves systematically coding text based on the words and language used.^36^, ^37^ Changes in each health behavior since becoming a caregiver were coded inductively using participants' words to generate codes. These codes were then compared across participants, and codes with a similar meaning were grouped together (e.g., ‘eating more takeaway’ was grouped with ‘eating more junk food’). Coding continued until no further codes could be extracted and the data could be sorted into existing codes without any discrepancies or overlap. For each change in health behavior identified, a second round of coding was undertaken to code the factors underlying the change in behavior, as described by the participant.

To identify targets for interventions to support the health and wellbeing of rural caregivers, the underlying factors identified were mapped to the socioecological framework.^38^, ^39^ The socioecological framework has been used in previous studies to identify factors influencing the health and wellbeing of population groups across multiple levels.^40^ The framework consists of five levels of influence: individual, interpersonal, organizational, community, and policy. For this analysis, the five levels were operationalized as follows: individual (caregivers' knowledge and skills), interpersonal (the caregiving role and social support), organizational (metropolitan‐based accommodation and treatment centers), community (living in a rural area), and policy (broader systems and policies).

### 2.4 Researcher characteristics and reflexivity

Most of the research team have previously or currently lived in a rural area of Queensland, Australia. Further, due to their client‐facing roles in a community cancer support organization, some have experience working closely with individuals living in rural areas. The research team is comprised of health behavior and cancer epidemiology researchers with qualifications in dietetics and psychology and experience conducting qualitative research, as well as an experienced cancer support advisor. We acknowledge that our underlying assumption in this analysis is that maintaining one's own health and wellbeing is a priority. The inherent value we place on optimal health and wellbeing is informed by our professional knowledge and experiences. However, we recognize that participants in this study may not share this value to the same extent or in the same way due to their own experiences. Indeed, as discussed later, our findings suggest that when caring for someone with cancer, rural caregivers may prioritize the health and wellbeing of their loved one over themselves. This highlights the importance of directly consulting with rural caregivers about their experiences, as per this study's aims and methods, to ensure that interventions designed to support their health and wellbeing align with their goals and values.

## 3 RESULTS

### 3.1 Participants

Key characteristics of the 20 rural caregivers included in this study are summarized in Table 1. Most caregivers were the spouse or partner (*n* = 13; 65%) of the person they were caring for, or another family member (*n* = 6; 29%) or neighbor (*n* = 1; 5%). Time since the patient's most recent cancer diagnosis ranged from 2 months to 10 years (median 3 years). This study included people caring for cancer survivors with a wide range of cancer types, including head and neck (*n* = 5; 25%), gynecological (*n* = 4; 20%), skin (*n* = 3; 15%), breast (*n* = 3; 15%), gastrointestinal (*n* = 2; 10%), brain (*n* = 1; 5%), blood (*n* = 1; 5%), and lymphoma (*n* = 1; 5%). Caregivers' ages ranged from 31 to 71 years, included males (*n* = 10; 50%) and females (*n* = 10; 50%), and all lived in an inner or outer regional area.

**TABLE 1:** Key characteristics of the 20 rural caregivers included in the content analysis of how caring for someone with cancer affects the health behaviors of rural caregivers.

ID	Relationship to person with cancer	Time since patient's most recent cancer diagnosis	Cancer type	Age bracket (years)	Geographical remoteness^a^
1	Neighbor (female)	6 months	Head and neck	60–69	Outer regional
2	Sister	4 months	Head and neck	40–49	Outer regional
3	Sister	Not reported	Stomach	60–69	Inner regional
4	Wife	5 years	Skin	40–49	Inner regional
5	Daughter	2 months	Head and neck	30–39	Outer regional
6	Husband	>12 months	Endometrial	60–69	Inner regional
7	Daughter	6 months	Ovarian	50–59	Inner regional
8	Partner (female)	3 months	Lymphoma	30–39	Inner regional
9	Husband	6 months	Ovarian	50–59	Inner regional
10	Mother	3 years	Cervical	60–69	Outer regional
11	Husband	Not reported	Skin	60–69	Outer regional
12	Partner (male)	3 years	Skin	60–69	Outer regional
13	Husband	3 years	Pancreas	50–59	Inner regional
14	Mother	Not reported^b^	Blood	60–69	Inner regional
15	Husband	4 years	Breast	50–59	Outer regional
16	Husband	3 years	Head and neck	50–59	Inner regional
17	Husband	4 years	Brain	40–49	Outer regional
18	Wife	10 years	Head and neck	50–59	Inner regional
19	Husband	7 years	Breast	60–69	Outer regional
20	Husband	8 years	Breast	70–79	Outer regional

^a^ Level of geographical remoteness was determined from postcode of residence using the Australian Statistical Geography Standard Remoteness Structure.

^b^ Patient was first diagnosed 21 years ago, has since experienced multiple cancer recurrences, and is currently undergoing active treatment.

### 3.2 Factors underlying changes to health behaviors of rural caregivers across the five levels of the socioecological framework

**FIGURE 1:**
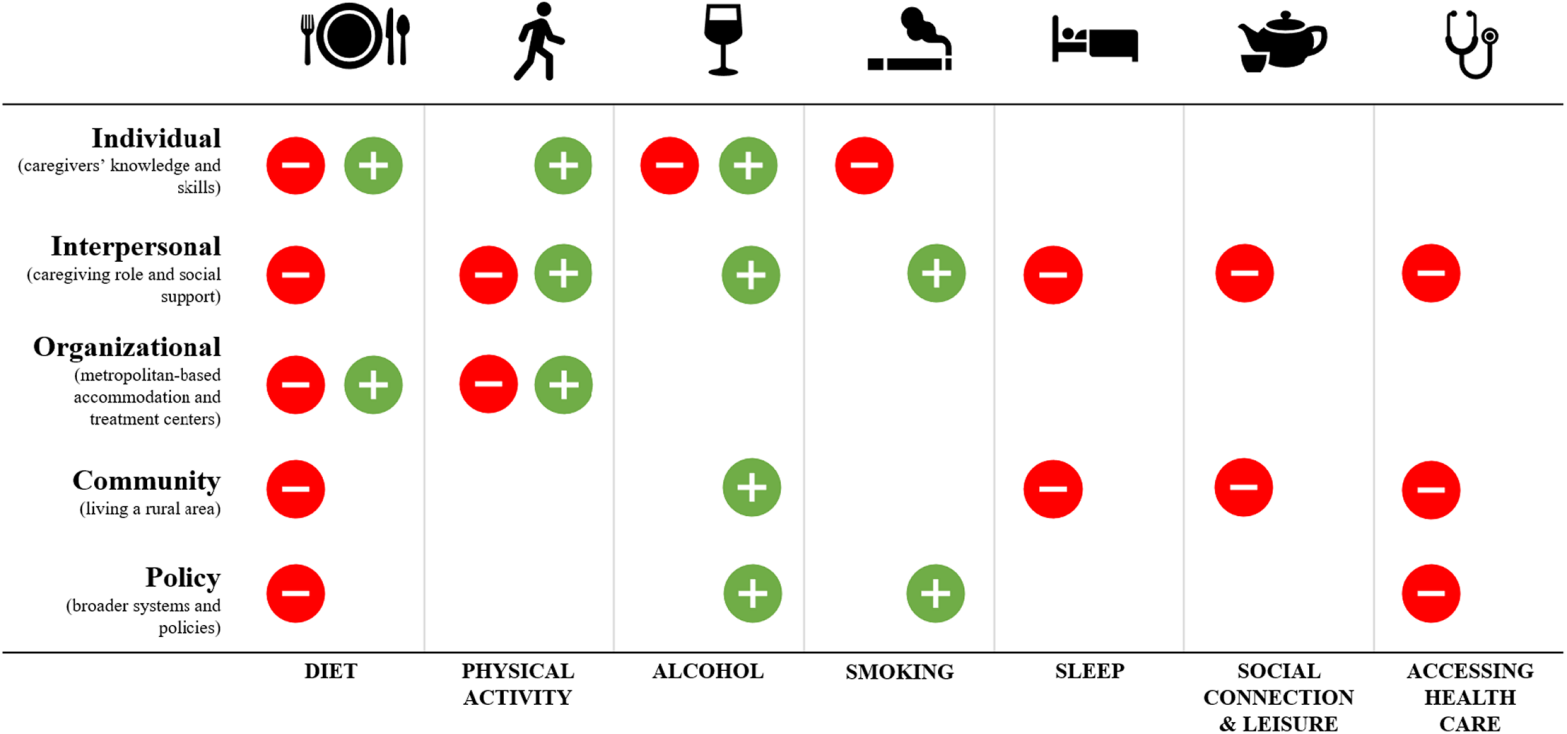
Summary of changes to rural caregivers' health behaviors by level of the socioecological framework.

All rural caregivers reported changes in more than one health behavior while caring for someone with cancer, including changes to their diet, physical activity, smoking, alcohol, sleep, social connection and leisure, and accessing health care when needed. The factors influencing these behavior changes are mapped across the five levels of the socioecological framework (individual, interpersonal, organizational, community, and policy) (see Figure 1 and Table 2).

**TABLE 2:** Factors underlying changes in rural caregivers' health behaviors while caring for someone with cancer mapped to the five levels of the socioecological framework.

Level	Factor	Health behavior	Change	Sample of participant quotes
Individual	1.1 Personal coping strategies	Diet	Increased intake of calorie‐dense foods	*“I might have ate a lot of chocolates and lollies*, *comfort food*.*”* (*C09*) *“I do eat more chocolate*. *That's my stress relief*.*”* (*C14*)
		Physical activity	Increased planned exercise	*“At the beginning it was probably less*, *but now I'm finding I'm walking heaps*. *I guess because I'm a bit more self‐aware and I can't sit still for too long […] I'd be gone for half an hour just going for a walk downstairs and coming back up […] It's the walk that keeps your mind healthy*, *even when you're at the hospital*.*”* (*C05*)
		Alcohol	Increased alcohol consumption	*“Drinking quite a lot [*…*] it's something to go away to*, *to release your mind*, *put it that way*.*”* (*C17*)
		Smoking	Increased cigarette use	*“I don't smoke often […] It's been tough*, *but I think I was just like*, *oh my god*, *I've been here for so long*. *Like*, *I just need to have a drink and a smoke*, *and I'm done*. *I can turn off straight away*.*”* (*C05*)
	1.2 Own health condition	Diet	Improved diet quality	*“Since I've got diabetes*, my eating has – I try and eat a lot healthier because I can't eat the s*** that I used to eat.*”* (*C10*)
		Alcohol	Reduced alcohol consumption	*“From time to time*, *I had a little bit more than usual*, *but with diabetes and all that*, *I had to cut down and be a bit more sensible*.*”* (*C16*)
Interpersonal	2.1 Demand of caregiving responsibilities	Diet	Reduced meal consistency	*“When I first got here with mum*, *I was with her what felt like 24/7 […] and I actually forgot to eat for at least the first 2 weeks […] I'd stop and I'd realize I was hungry*, *and I thought*, *I need to have something to eat*. *It would just be like one o'clock and I'd look up and just kind of realize*, *I haven't eaten since breakfast*.*”* (*C05*)
			Increased convenience food/takeaway consumption	*“If I stay up with mum until 7 or 8 at night*, *I know I can just nip across […] and grab a burger [*…*] It's just not as many home‐cooked meals*.*”* (*C05*) *“I think we ate more takeaway than anything because you can't cook as much when you're away from home*, *you know? Because you've got appointments to go to […] like when you're getting radiation*, *it may be in the afternoon or the morning*, *it just depends*.*”* (*C18*)
		Physical activity	Reduced planned exercise	*“I'm still active around the house doing stuff and that but well before she got sick […] we walked to the beach from where we are and I was swimming every second morning […] but when she got sick*, *that sort of disrupted my schedule*.*”* (*C06*)
			Increased incidental exercise	*“We're pretty active because every day there's always something going on*. *Like we've always got to be at the hospital or the physio or the podiatrist […] all the appointments and everything*.*”* (*C08*)
		Alcohol	Reduced alcohol consumption	*“I was very surprised that I didn't*, *you know*, *go out and drink myself silly because of it*. *But yeah*, *there was too much else going on because I just didn't have anyone there to help me*.*”* (*C09*) *“It wasn't like we turned to alcohol to get through the disruption and pain*. *If anything*, *we slowed down in that period*, *I think because we were dealing with everything else*.*”* (*C19*)
		Sleep	Reduced sleep quality / quantity	*“I've got things to do*. *Things to organize and I haven't had sleep yet*.*”* (*C02*)
		Accessing health care	Delays in accessing health care	*“That was sort of the exception that you'd have to make*. *There were things that I sort of put off or delayed until a later date*. *My main priority was just to get through what we were dealing with*.*”* (*C19*)

	2.2 Prioritizing the patient's needs	Diet	Changes to type of food consumed	*“We're on a high energy*, *high fiber*, *high protein diet*. *So yeah*, *it's a lot different […] I just eat whatever [my partner] eats*, *it's easier*.*”* (*C08*)
			Reduced meal consistency and portions	*“I don't eat as much as I used to*. *If [my wife] doesn't eat*, *I can't eat [*…*] Portions have gone down*.*”* (*C13*)
			Increased intake of calorie‐dense foods	*“Because of her medication and the chemo*, *[patient] has had a want of things that are very oily and fatty like fish and chips […] because the chemo has affected her senses […] so for a time*, *at least a couple of years*, *we did consume a lot of things that weren't that healthy like chips and goodies and lollies and fatty things like takeaway*.*”* (*C15*).
		Physical activity	Reduced planned exercise	*“That would all depend on how [my wife] was feeling*, *especially when she was going through the treatment of chemo or radiation*. *If she wasn't well*, *I wouldn't go to squash*.*”* (*C20*)
Social connection and leisure		Reduced social connection	*“I must admit that the fact that [my partner] is in a lot of pain [*…*] she doesn't want to socialize too much [*…*] It limits my ability to socialize also*, *because I want to go with her*. *And now that's just a realization that we've started to come to just recently is that I have to do things also on my own*.*”* (*C15*) *“We belonged to a motorcycle social club and we used to go right around the countryside*, *and we don't do that anymore*. *[My wife] doesn't like the idea of being on the bike and feeling the seat so we don't do that much*.*”* (*C13*)
Interpersonal	2.3 Carer fatigue and distress	Diet	Reduced meal consistency	*“I just wouldn't eat […] I would be that exhausted when I got into the shower that I would forget to brush my teeth […] at the time I wasn't […] getting domestic care […] we weren't even eating properly*.*”* (*C04*) *“At times I couldn't be bothered cooking myself a proper meal […] I'd always have something for my son*, *but I'd just skip a meal or just have a small meal because I didn't really feel like eating*.*”* (*C20*)
			Increased convenience food/takeaway consumption	*“You spend a lot of time in hospital and by the time you leave the hospital*, *oh*, *I'll just get a burger from across the road and that'll be my tea […] even if you have got the food back at the hospital*, *it's exhausting*, *draining sitting around at hospital all the time*.*”* (*C14*)
		Smoking	Reduced cigarette use	*“When I am absolutely exhausted*, *that's when my smoking will actually slow right down […] I just don't have the energy to do anything*.*”* (*C04*)
		Sleep	Reduced sleep quality / quantity	*“Not much sleep*. *Broken sleep*. *Just take some medication and have a couple [pain killers] […] and have a couple of hours sleep and constantly be watching the phone in case something's going on*.*”* (*C09*)
		Social connection and leisure	Reduced social connection	*“I found that I wasn't answering the phone and I wasn't leaving the house*, *because if I run into people they'd say*, *how are you? How's [your daughter]? And then*, *that's it*, *I'd just start crying*. *So*, *for about 3 weeks*, *I just shut myself away*.*”* (*C14*) *“All mine and [my partner's] friends are back home and we do have quite a lot of friends up here*, *but with the COVID wave and everyone's sick at the moment*, *we can't really see anybody […] you can't go to the pub or catch up with people for too long because you don't want [my partner] to be susceptible to getting sick or anything […] It does get a little bit lonely*.*”* (*C08*)
	2.4 Support from friends at home	Physical activity	Increased planned exercise	*“Since I've been back at home this last couple of months […] my best mates have been getting me out and we've been doing a bit of walking and playing golf again and that sort of stuff*.*”* (*C10*)
Organizational	3.1 Location of accommodation in major cities	Diet	Reliance on convenience foods	*“We were staying at [the lodge]*, *and as you're probably aware*, *there's nothing around [*…*] I had to find bread and milk and all that's around is that service station across from the hospital and they sure know how to charge you [*…*] And then I just had to eat stuff from the cafeteria*.*”* (*C14*)
		Physical activity	Increased incidental exercise	*“Now I find I'm walking heaps […] Since being at the lodge*, *the bus only runs at certain times from here […] I just figured out which bus station was closer*, *and I walked myself down to the bus station*.*”* (*C05*) *“[The] lodge is a couple of blocks away from the hospital and so I would walk [*…*] there was a bus from [the lodge] but not at the times for me*.*”* (*C14*)
	3.2 Access to facilities while staying in major cities	Diet	Maintained nutritional value of meals	*“I'm trying to keep fairly strictly to the sort of food I eat*. *Not takeaways because I don't normally eat takeaways anyway but yeah*, *my diet is very similar*. *I was in a lovely unit that had a kitchen*, *a full kitchen*, *and um*, *that was just amazing*, *but this has lingered on longer so now I've been moved to a little tiny unit with the kitchen nearby*, *but my needs are fairly simple*, *and I can easily cook up food and eat healthily*.*”* (*C03*) *“We were able to cook in the hospital […] and so we would cook a meal in the hospital in the parent's kitchen and we would all […] sit around [my daughter's] bed and eat as a family like we do at home*.*”* (*C14*)
			Increased nutritional value of meals	*“Where we were living*, *it wasn't a good situation […] it was mainly takeout and meals that could be quickly prepared and stuff like that*. *But yes*, *since we've moved to the lodge*, *we've been eating a lot better […] we're close to the communal kitchen*, *which is really good*.*”* (*C10*)
		Physical activity	Reduced planned exercise	*“I have an electric bike at home*, *and I can't walk far*. *I stretch as much as I can and do the steps*, *stretch a bit*, *go for a walk but not far*. *If I had my bike*, *it'd be good*. *I use my electric bike*.*”* (*C11*)
Community	4.1 Need to travel for treatment	Diet	Increased convenience food / takeaway consumption	*“You can only get what's available*. *Just the convenience store on the end of the corner when you first get to [the city] […] The timing for plane trips and all that*. *You grab what you can […] And when you're at home*, *you don't feel like cooking anyway because you've got to go buy groceries*. *You know you're going to be on a plane again leaving it*.*”* (*C01*)
		Alcohol	Reduced alcohol consumption	*“I didn't drink any alcohol for about probably 6 months while I was [in the city] and that was very strange for me*, *because I do play golf*. *I was playing golf Saturday*, *Sunday every week and I would have quite a few beers*, *but that was the one thing I didn't do*. *I didn't drink*.*”* (*C09*)
		Sleep	Reduced sleep quality / quantity	*“That affects your sleep*. *You're always on high alert*. *Packing and unpacking […] so lovely long sleeps are sort of out of the question because you wake up in the middle of the night*. *Did I remember this? Did I remember that?”* (*C01*).
		Social connection and leisure	Reduced social connection	*“I was pretty isolated during the main part of the treatment*. *We were keeping to ourselves and then in between trips*, *sort of building up to the next round of treatment*.*”* (*C16*) *“I didn't have friends or family in [the city] […] but they always kept in contact with me*. *My friends rang me every day […] [but] when you haven't got someone there to give you some support*, *give you some help*, *[except] over the phone*, *it's a little bit difficult*.*”* (*C09*)
		Accessing health care	Reduced access to health care	*“So*, *it's very hard to get a doctor's appointment […] because if I get a doctor's appointment and then I have to fly out*. *Oh*, *sorry*, *I've got to cancel*, *you know*.*”* (*C01*)
Policy	5.1 Costs of caregiving and travel	Diet	Unable to afford basic food items	*“And even just like bread and milk*. *You pay a fortune just for a liter bottle of milk at the service station and I sort of think*, *oh*, *do I really need it? That's quite expensive*. *A one liter bottle of milk at the servo is the cost of the cheapest 2 liter you can get at the supermarket [back home]*. *Yeah*, *$2*.*10 for a 2 liter and then it was like $2*.*80 for a one liter*. *And you think*, *I don't want to pay that*, *that's robbery!”* (*C14*)
		Alcohol	Reduced alcohol consumption	*“I don't drink as much at all […] I was on that unemployment benefit*, *so I only had that meager income*. *And it took 6 months for Centrelink to put me on to the carer pension […] so*, *for the whole 6 months doing everything*, *I didn't have time to go buy beer*. *I didn't have the money to buy beer*.*”* (*C01*)
		Accessing health care	Unable to afford own health care	*“I couldn't afford [medical care]*. *That's what I say to [my partner]*, *we can't afford me to get sick because it's costing me a fortune to keep her*. *Getting her back in health and everything like that*. *There's two or three things I've got to start looking at too*, *before they get too big or too broken*.*”* (*C12*)
	5.2 Public health policies	Smoking	Reduced cigarette use	*“I smoke less because you've got to go for a walk to have a smoke […] Whenever I sometimes feel like a cigarette because I've got to go for a walk around the block*, *if I don't feel like going for a walk*, *I just have something to eat*.*”* (*C08*)

### 3.3 Factors at the individual level

#### 3.3.1 Personal coping strategies

The caregiver's personal coping strategies and health condition contributed to changes in their health behaviors while caring for someone with cancer. Personal coping strategies for dealing with a loved one's cancer diagnosis impacted diet, physical activity, alcohol, and smoking (Table 2, Factor 1.1). For example, rural caregivers reported increased intake of calorie‐dense food related to comfort and emotional eating, increased intentional exercise to manage restlessness while spending long hours at the hospital, and increased use of cigarettes and alcohol, described by caregivers as a way to “turn off” or “release your mind.”

#### 3.3.2 Own health condition

Some caregivers reported that while caring for someone with cancer, they were also managing a chronic condition of their own, such as diabetes, mental illness, and sleep apnea. Some caregivers reported making healthier food choices and reducing their alcohol consumption to look after their own health so that they could care for their loved one with cancer (Table 2, Factor 1.2).

### 3.4 Factors at the Interpersonal level

#### 3.4.1 Demand of caregiving responsibilities

The demands of caregiving responsibilities, prioritizing the patient's needs, carer fatigue and distress, and support from friends at home affected rural caregivers' health behaviors. Caregiving roles required a significant amount of time, and the additional responsibilities negatively affected caregivers' diet, physical activity, sleep, and accessing health care when needed (Table 2, Factor 2.1). Rural caregivers reported reduced meal consistency, higher intake of convenience foods, poorer sleep, and delaying accessing health care for themselves due to the constancy of care required by the patient. Some caregivers reported less planned exercise from disruptions to daily routines while others described increased incidental exercise from traveling to the various medical appointments. On a positive note, the additional responsibilities and organization required meant that, for some caregivers, there was less time for alcohol consumption, and they had subsequently lowered their intake while caring for someone with cancer.

#### 3.4.2 Prioritizing the patient's needs

Rural caregivers often spoke of prioritizing the patient's needs above their own, impacting their diet, physical activity, social connection, and leisure (Table 2, Factor 2.2). This included changing the type of food they consumed, reduced meal consistency and portions, and increased their intake of calorie‐dense foods to accommodate the patient's dietary needs. Caregivers also reported reducing their physical activity and socialization in response to the patient's lower energy levels and physical capacity.

#### 3.4.3 Carer fatigue and distress

Fatigue and psychological distress from the caregiving role contributed to negative changes in diet, smoking, sleep, social connection, and leisure (Table 2, Factor 2.3). Caregivers spoke about being too exhausted to prepare or eat “a proper meal” and consuming more convenience food items and takeaway. Some reported reduced sleep quality and quantity due to distress and worry. Exhaustion and distress reduced use of not only the use of cigarettes but also socialization, as caregivers avoided opportunities for social interaction due to the emotional toll of others asking after themselves and their loved ones.

#### 3.4.4 Support from friends at home

Finally, friends in the caregivers' local community who organized social activities to support the caregiver had a positive impact on their physical activity (Table 2, Factor 2.4).

### 3.5 Factors at the organizational level

#### 3.5.1 Location of accommodation in major cities

The location of accommodation while staying in a major city for the patient's cancer treatment and access to facilities while away from home influenced rural caregivers' health behaviors. Just over half of caregivers in this study stayed at one of the CCQ accommodation lodges, and its suburban location affected their diet and physical activity (Table 2, Factor 3.1). Caregivers reported difficulty accessing grocery stores while staying at the accommodation lodges, often requiring a taxi or bus trip to purchase groceries. This affected their diet through their reliance on convenience stores nearby. However, caregivers did report increased incidental exercise as the proximity of the lodge to the hospital meant they could walk there to visit the patient.

#### 3.5.2 Access to facilities while staying in major cities

Access to facilities while staying in a major city influenced caregivers' diet and physical activity (Table 2, Factor 3.2). This included being able to prepare their own meals using the cooking facilities within their units, as well as the communal kitchen at the accommodation lodge or at the hospital while visiting the patient. This reduced caregivers' reliance on takeaway and convenience foods. However, caregivers did report less planned exercise while away from home due to not having access to their usual exercise gear and equipment.

### 3.6 Factors at the community level

#### 3.6.1 Need to travel for treatment

Living in a rural area and the resultant need to travel to a major city for treatment had a widespread impact on rural caregivers' health behaviors. Traveling for treatment negatively affected rural caregivers' diet, sleep, social connection and leisure, and accessing health care when needed (Table 2, Factor 4.1). Rural caregivers reported increased intake of convenience food items and takeaway meals, especially while waiting in airports and upon arriving in an unfamiliar city. Even when at home, caregivers reported increased use of convenience food items and takeaway meals as the frequent travel for cancer treatment or follow‐up meant that groceries and food prepared were often not consumed before needing to travel again. The regular travel also disrupted caregivers' sleep and their ability to spend time with friends or book medical appointments ahead of time. On the other hand, being away from home meant that some caregivers consumed less alcohol due to not engaging in their usual social weekend activities.

### 3.7 Factors at the policy level

#### 3.7.1 Cost of caregiving and travel

The cost of caregiving and traveling for treatment, as well as policies for public health, affected rural caregivers' health behaviors. For many caregivers, the costs of caring for someone with cancer, including having to stop work and higher living expenses while away from home, affected their diet, alcohol consumption, and ability to access health care when needed (Table 2, Factor 5.1). Rural caregivers reported being unable to afford basic food items in major cities and medical care for themselves. One positive outcome from the financial impact of caregiving was less alcohol consumption due to reduced purchase of discretionary items.

#### 3.7.2 Public health policies

Finally, for rural caregivers who would normally smoke throughout the day, the no smoking policy at the hospital meant they were not able to smoke as often as usual, resulting in reduced cigarette use while caring for someone with cancer (Table 2, Factor 5.2).

## 4 DISCUSSION

This study examined changes in rural caregivers' health behaviors while caring for someone with cancer and the socioecological factors (i.e., factors at the individual, interpersonal, organizational, community, and policy levels) that influenced these changes. Rural caregivers reported both positive and negative changes to their diet, physical activity, alcohol, and smoking, whereas sleep, social connection and leisure, and accessing health care when needed were consistently negatively impacted. Importantly, the socioecological factors affecting rural caregivers' health behaviors were not unique to specific health behaviors; rather, these factors influenced multiple behaviors. Therefore, designing interventions to address these underlying factors, as well as specific health behaviors, could yield widespread benefits for rural caregivers' health and wellbeing. Across all levels, from individual to policy, it will be important for supportive care interventions to be co‐designed with rural cancer caregivers, as interventions that are designed in collaboration with end users have been shown to be more acceptable, accessible, and translatable beyond the research setting.^41^

Many of the changes to health behaviors identified in this study have been reported in previous studies of cancer caregivers in general.^20^, ^21^, ^22^ However, because our study examines the factors underlying changes in health behaviors and is specific to rural caregivers, we were able to identify opportunities for supporting the health and wellbeing of people who need to travel long distances for cancer treatment and who rely on the facilities available at their accommodation or hospital while away from home. These factors had a particularly negative impact on their diet, sleep, social connection and leisure, and accessing healthcare when needed.

### 4.1 Implications for policy and practice

Findings from this study indicate that interventions to improve rural caregivers' health and wellbeing are needed across multiple levels. At the individual level, our research suggests that, for some caregivers, maladaptive coping strategies in response to stress may contribute to poorer health behaviors, such as comfort eating and increased use of alcohol and cigarettes. Therefore, equipping rural caregivers with effective coping strategies might mitigate these changes. For example, web‐based interventions that include an online support group, with or without informational materials, have been shown to significantly improve coping skills among cancer caregivers.^42^ Such interventions could be accessed by rural caregivers at home or while traveling for treatment and may also provide opportunities for social connection, improving other aspects of their health and wellbeing.^43^ However, it will also be important to consider the accessibility and acceptability of digitalized interventions for people in rural areas, including strategies to support digital infrastructure and literacy.^44^

At the interpersonal level, interventions are needed to reduce caregiver burden, exhaustion, and stress. Based on our findings, these interventions could include practical support, such as simple ideas for meal preparation, strategies for relaxation and rest, or opportunities for caregiver respite. Encouraging caregivers to prioritize their own health and wellbeing may be important since rural caregivers reported prioritizing the patient's needs above their own, with negative impacts on their diet, physical activity, social connection, and leisure. Barriers to accessing support for their own health and wellbeing may also need further investigation. Previous research among rural caregivers identified many who do not seek support for themselves while caring for someone with cancer^45^ and reluctance to use professional emotional support due to the time required and potential discomfort.^28^ It is not known how a decline in health behaviors while caring for someone with cancer affects caregivers' long‐term health outcomes, particularly where caregivers' support extends well beyond initial diagnosis and treatment phases.

At the organizational and community levels, multiple health behaviors were affected by the disruption to usual routine caused by regular travel, as well as staying in an unfamiliar environment. Our findings suggest that minimizing the need to travel for treatment is a priority for supporting the health and wellbeing of rural caregivers. This could be achieved by coordinating appointments between healthcare professionals in metropolitan‐based centers or providing follow‐up via telehealth where possible.^46^

At the policy level, further work is needed to improve accessibility to welfare support for carers, including prompt eligibility assessments and payments. This need has been expressed by rural caregivers in other studies,^47^ and our findings suggest that improving access to welfare support may benefit rural caregivers' diet, social connection and leisure, and use of health care services when needed. This welfare support includes both unemployment and carer benefits, as well as travel subsidies. For caregivers in Queensland, the patient travel subsidy scheme (PTSS) recently increased to $70 AUD per night for accommodation and $0.34 AUD per kilometer of travel in a private car.^48^ However, media reports indicate that these amounts are still too low to alleviate the financial burden of traveling for treatment, with cancer patients and their carers reporting significant out‐of‐pocket costs and financial strain.^49^

### 4.2 Strengths and limitations

A key strength of this study is the diverse sample of rural caregivers, including different cancer types, relationships, age, and time since diagnosis. However, none of the caregivers lived in remote areas. While the changes identified in this study may also be experienced by those caregivers, there may be additional changes in health behaviors and factors underlying those changes that are not captured in this analysis. While some caregivers spoke about their mental health in relation to the impact of caregiving on their health behaviors, future research could examine this further, particularly since rural caregivers report high levels of psychological distress compared to people of the same age in the general population.^18^ Finally, the qualitative approach to this analysis gives rural caregivers a ‘voice’, with participant quotes used to describe changes in health behaviors and factors underlying these changes. Future research using longitudinal, population‐wide surveys could investigate absolute change in health behaviors and the frequency or prevalence of change.

## 5 CONCLUSION

Caring for someone with cancer has a widespread impact on rural caregivers' health behaviors. Rural caregivers experienced both positive and negative changes to their diet, physical activity, alcohol consumption, and cigarette use. Sleep, social connection and leisure, and use of health care services were negatively affected due to the demands of caregiving responsibilities and the need to travel for treatment. To support the health and wellbeing of rural caregivers, interventions across multiple levels are needed, including individual, interpersonal, organizational, community, and policy levels. This could include equipping caregivers with effective coping strategies for stress, practical support to reduce caregiver burden and fatigue, encouraging caregivers to prioritize their own needs and seek support, access to cooking and exercise facilities and social support while away from home, reducing the need to travel for treatment or follow‐up, and increasing financial support available to carers. Interventions that focus on addressing these factors are likely to benefit multiple health behaviors for those living in a rural area and caring for someone with cancer.

## AUTHOR CONTRIBUTIONS

**Elizabeth A. Johnston:** Conceptualization (equal); formal analysis (equal); investigation (lead); project administration (lead); resources (supporting); supervision (equal); visualization (equal); writing – original draft (equal); writing – review and editing (equal). **Katelyn E. Collins:** Data curation (lead); formal analysis (equal); visualization (equal); writing – original draft (equal); writing – review and editing (equal). **Jazmin N. Vicario:** Data curation (supporting); formal analysis (supporting); resources (supporting); writing – original draft (equal); writing – review and editing (equal). **Chris Sibthorpe:** Resources (lead); writing – review and editing (equal). **Michael J. Ireland:** Supervision (equal); writing – review and editing (equal). **Belinda C. Goodwin:** Conceptualization (equal); writing – review and editing (equal).

## FUNDING INFORMATION

None.

## CONFLICT OF INTEREST STATEMENT

The authors declare that there is no conflict of interest.

## ETHICAL APPROVAL

Ethical approval for this study was granted by the Human Research Ethics Committee of the University of Southern Queensland (H17REA152).

## Supporting information


Tables S1–S2.

## Data Availability

The data that support the findings of this study are available on request from the corresponding author. The data are not publicly available due to privacy or ethical restrictions.
