# Supplementary material for: Changes in rural caregivers' health behaviors while supporting someone with cancer: A qualitative study
Source: Cancer Med. 2024 Apr 4;13(7):e7157. doi: 10.1002/cam4.7157 (PMC10993705; doi:10.1002/cam4.7157)
Supplement: Supplementary file 1 — Tables S1–S2. [file CAM4-13-e7157-s001.docx]

**Supplementary Table 1.** Standards for Reporting Qualitative Research (SRQR).

O’Brien B.C., Harris, I.B., Beckman, T.J., Reed, D.A., & Cook, D.A. (2014). Standards for reporting qualitative research: a synthesis of recommendations. *Academic Medicine, 89(9)*, 1245-1251.

| **No. Topic** | **Item** | **Addressed on pg #** |
| --- | --- | --- |
| **Title and abstract** |  |  |
| S1 Title | Concise description of the nature and topic of the study identifying the study as qualitative or indicating the approach (e.g., ethnography, grounded theory) or data collection methods (e.g., interview, focus group) is recommended | 1 |
| S2 Abstract | Summary of key elements of the study using the abstract format of the intended publication; typically includes objective, methods, results, and conclusions | 3 |
| **Introduction** |  |  |
| S3 Problem formulation | Description and significance of the problem/phenomenon studied; review of relevant theory and empirical work; problem statement | 4-5 |
| S4 Purpose or research question | Purpose of the study and specific objectives or questions | 5 (lines 71-78) |
| **Methods** |  |  |
| S5 Qualitative approach and research paradigm | Qualitative approach (e.g., ethnography, grounded theory, case study, phenomenology, narrative research) and guiding theory if appropriate; identifying the research paradigm (e.g., positivist, constructivist/interpretivist) is also recommended | 7 (lines 120-123) |
| S6 Researcher characteristics and reflexivity | Researchers’ characteristics that may influence the research, including personal attributes, qualifications/experience, relationship with participants, assumptions, or presuppositions; potential or actual interaction between researchers’ characteristics and the research questions, approach, methods, results, or transferability | 8 (lines 142-153) |
| S7 Context | Setting/site and salient contextual factors; rationale^a^ | 6 |
| S8 Sampling strategy | How and why research participants, documents, or events were selected; criteria for deciding when no further sampling was necessary (e.g., sampling saturation); rationale^a^ | 6 |
| S9 Ethical issues pertaining to human subjects | Documentation of approval by an appropriate ethics review board and participant consent, or explanation for lack thereof; other confidentiality and data security issues | 6 (lines 97-99) |
| S10 Data collection methods | Types of data collected; details of data collection procedures including (as appropriate) start and stop dates of data collection and analysis, iterative process, triangulation of sources/methods, and modification of procedures in response to evolving study findings; rationale^a^ | 7 |
| S11 Data collection instruments and technologies | Description of instruments (e.g., interview guides, questionnaires) and devices (e.g., audio recorders) used for data collection; if/how the instrument(s) changed over the course of the study | 7 |
| S12 Units of study | Number and relevant characteristics of participants, documents, or events included in the study; level of participation (could be reported in results) | 9 |
| S13 Data processing | Methods for processing data prior to and during analysis, including transcription, data entry, data management and security, verification of data integrity, data coding, and anonymization/deidentification of excerpts | 7-8 |
| S14 Data analysis | Process by which inferences, themes, etc., were identified and developed, including researchers involved in data analysis; usually references a specific paradigm or approach; rationale^a^ | 7-8 |
| S15 Techniques to enhance trustworthiness | Techniques to enhance trustworthiness and credibility of data analysis (e.g., member checking, audit trail, triangulation); rationale^a^ | 7, lines 130-132 |
| **Results/Findings** |  |  |
| S16 Synthesis and interpretation | Main findings (e.g., interpretations, inferences, and themes); might include development of a theory or model, or integration with prior research or theory | 9-13 |
| S17 Links to empirical data | Evidence (e.g., quotes, field notes, text excerpts, photographs) to substantiate analytic findings | 27 (table 2) |
| **Discussion** |  |  |
| S18 Integration with prior work, implications, transferability, and contribution(s) to the field | Short summary of main findings; explanation of how findings and conclusions connect to, support, elaborate on, or challenge conclusions of earlier scholarship; discussion of scope of application/generalizability; identification of unique contribution(s) to scholarship in a discipline or field | 14-16 |
| S19 Limitations | Trustworthiness and limitations of findings | 16 |
| **Other** |  |  |
| S20 Conflicts of interest | Potential sources of influence or perceived influence on study conduct and conclusions; how these were managed | N/A |
| S21 Funding | Sources of funding and other support; role of funders in data collection, interpretation, and reporting | N/A |

**Supplementary Table 2.** Semi-structured interview guide for examining changes in rural caregivers’ health behaviors since becoming a caregiver and factors underlying these changes.

| **Content area** | **Question** | | **Prompts / further questions** |
| --- | --- | --- | --- |
| Cancer burden | 1. To start, can you tell me a bit about [person’s] cancer diagnosis? | | What type of cancer were they diagnosed with? Do you know what stage – did they find it early or was it a bit more advanced? What type of treatments have they had? What side-effects have they experienced from their cancer / treatment? |
|  | 1. Where are they now in their cancer journey? | | Are they currently receiving treatment or finished treatment? Do you know if the cancer has responded to the treatment? |
| Support role | 1. In what ways do you support [person]? | | What activities do you help with? How long have you been helping with these things? Are you the main support person / do others help out? Has supporting your [person] with cancer involved any changes to your employment? How has your support role changed since their diagnosis? |
| Health behaviours | 1. The next few questions are about how supporting [person] with cancer has affected your health behaviours. It often helps to think of them one at a time, so let’s start with diet.    1. Has supporting [person] through their cancer journey affected what you eat, how and when you eat, that sort of thing? In what ways?    2. What about physical activity?    3. Getting enough sleep?    4. This isn’t relevant to everyone, but can I ask whether you normally smoke cigarettes or tobacco?    5. What about alcohol?    6. Finding time to relax / catching up with friends / hobbies?    7. Seeing a doctor when you need to, for example, if you have health problems of your own? | | Is there anything about the area you live in that affects [health behaviour]?  [If health behaviour has changed] Have these changes to your [health behaviour] affected your health and how you feel? In what ways?  Are there any other ways that supporting [person] with cancer has affected your ability to stay healthy and look after your own health? |
| Caregiver support | 1. This might seem obvious based on the previous questions, but we just ask everyone – Overall, do you feel you are able to balance caring for [person] with looking after your own health and wellbeing? | | Is there anything or anyone that has helped you to look after your own health and wellbeing while supporting [person]? Is there anything or anyone that would have been helpful in helping you to look after your own health and wellbeing while supporting [person]? Is this kind of support available in your local community? Do you have any recommendations for how other people who are living in rural areas and supporting someone with cancer can look after their own health and wellbeing?  **Prompts** – Friends or family? Doctor or other health professional? Information or support services? |
|  | 1. What could organisations like Cancer Council Queensland do to help you look after your own health and wellbeing while supporting [person]? | |  |
| Final questions | 1. So lastly, I just have two questions so we can describe the people we interviewed.    1. If you don’t mind, can I ask, what is your age in years?    2. And what is your residential postcode? |  | |

*Thank you for all the valuable information you have provided today. Do you have any other final comments about your health and wellbeing since [person’s] cancer diagnosis?*

*Well, if there’s ever a time that you feel you need further support or you’d just like to talk to someone about it, Cancer Council has a free and confidential telephone service. The number is 13 11 20. They can help you with getting cancer-related information and support. We appreciate your generosity to share your perspective, especially given all that’s going on for you. All the best to you and [person].*

*(Note in case: 13 11 20 open Monday to Friday, 9am to 5pm. After hours support available from Lifeline 13 11 14).*
